# Stroke Prevention in Atrial Fibrillation: Concepts and Controversies

**DOI:** 10.2174/157340312803760820

**Published:** 2012-11

**Authors:** Yousif Ahmad, Gregory YH Lip

**Affiliations:** University of Birmingham Centre for Cardiovascular Sciences, City Hospital, Birmingham, UK

**Keywords:** Atrial fibrillation, stroke prevention, oral anticoagulants.

## Abstract

Atrial fibrillation (AF) is the commonest cardiac rhythm disorder worldwide, affecting 1% of the general population. It is estimated that up to 16 million people in the US will suffer from the arrhythmia by 2050. AF is an independent stroke risk factor and associated with more severe strokes. For six decades, warfarin has been the only truly effective therapy to protect against stroke for patients with atrial fibrillation. Despite the proven worth of warfarin, its limitations have seen reluctance amongst physicians and patients to utilise this efficacious agent. This has meant that substantial numbers of patients are either unprotected against stroke or suboptimally protected with antiplatelet therapy.

Contemporary well-validated stroke risk factor schemes (CHA_2_DS_2_-VASc) now permit rapid but comprehensive evaluation of a patient’s risk for thromboembolism, allowing better identification of low-risk patients who do not require antithrombotic therapy, and whilst for those with ≥1 stroke risk factors require formal oral anticoagulation. Aspirin has been proven to be inferior to anticoagulation, and is not free of bleeding risk. We also have simple scores to easily evaluate a patient’s risk of haemorrhage (e.g. HAS-BLED).

The emergence of new oral anticoagulants should further improve stroke prevention in AF, and they successfully negotiate many of the hurdles to oral anticoagulation generated by warfarin’s limitations. Monitoring, reversal, and perioperative management are areas which require further investigation to enhance our ability to safely and effectively utilise the new agents.

## INTRODUCTION

Atrial fibrillation (AF) is the commonest cardiac rhythm disorder worldwide, affecting 1% of the general population [1]. Its prevalence increases with age [2,3], and 10% of those aged over 80 years are affected [1]. AF is increasing in prevalence and incidence, and it is estimated that up to 16 million people in the US will suffer from the arrhythmia by 2050 [4]. These increases are being driven by the ageing population and the increased survival of patients from both acute and chronic cardiac disorders which predispose to AF [5, 6]; the presence of AF in patients with underlying cardiac disease is associated with worse outcomes [7]. AF is found in roughly 5% of acute medical admissions [8] and the increased risk of morbidity and mortality associated with AF leads to a heavy public health burden and increased healthcare costs [9, 10]. 

AF leads to a prothrombotic state [11] which predisposes to stroke, the most devastating and most common complication of thromboembolism. AF is an independent risk factor for stroke and held responsible for 25% of all strokes [12]. The presence of AF increases the risk of stroke five-fold [13], and is also a risk factor for stroke recurrence [14]. As well as increasing the likelihood of a stroke occurring, AF is also associated with more severe strokes [15]. Patients with AF who suffer a stroke are more likely to die, spend longer in hospital, are more likely to be discharged to a nursing home placement and have a greater level of disability [16]. 

For six decades, vitamin K antagonists (i.e. warfarin) have been the only truly effective therapy to protect against stroke for patients with atrial fibrillation. Despite the proven worth of warfarin as thromboprophylaxis in AF, its limitations and inconveniences have seen reluctance amongst physicians and patients to utilise this efficacious agent [17]. This has meant that substantial numbers of patients are either unprotected against stroke or suboptimally protected with antiplatelet therapy – despite evidence suggesting that most thromboembolic complications could be avoided with anticoagulation [18]. 

The last decade has been an intensive period of research in AF which has finally seen the emergence of novel oral anticoagulants to complement and compete with warfarin. There has also been evidence to highlight the limitations of aspirin as thromboprophylaxis in AF, and improved risk stratification schemata to allow for the identification of patients truly at low-risk for thromboembolism and simple assessment of the bleeding risk. These advancements should combine to reduce the variability of management in AF stroke prevention and lead to more patients receiving anticoagulation to protect them against thromboembolism. The new agents, although overcoming many of warfarin’s limitations, will present their own set of challenges and considerations as they are incorporated into clinical practice.

## RISK STRATIFICATION

Although AF is an independent risk factor for stroke, this risk is not homogenous and depends on the presence or absence of specific risk factors for stroke in AF [19, 20]. These risk factors were incorporated into the simple CHADS_2_ score [21], which is the most widely used stroke risk factor scheme (see Table **1**). Its original validation categorised patients as “low-risk” if they scored 0, “intermediate-risk” if they scored 1-2 and “high-risk” if they scored 3 or higher. 

There are several drawbacks associated with this scoring schema. Older guidelines used this grouping to recommend oral anticoagulation to high-risk patients, aspirin for low-risk patients, and a choice of either anticoagulation or aspirin for the intermediate grouping. Thus a patient with a previous stroke could be classified as “intermediate risk” and be given aspirin in preference to warfarin. An analysis of hospital inpatients with AF [22] found that those deemed low risk by CHADS_2 _had a rate of thromboembolism of 1.67 per 100 person years, and those deemed at intermediate risk had a rate of 4.75 per 100 person years. The CHADS_2_ score characterises 65% of patients into the “intermediate” risk grouping [23]. This has the potential of causing confusion, as the guidelines did not give a clear instruction as to whether aspirin or warfarin should be given in this group of patients. There is evidence to suggest aspirin does not reduce the risk of stroke in low-risk patients [24] and that warfarin is superior to aspirin for patients deemed at moderate or intermediate risk [25]. Thus reliance on the CHADS_2_ score alone would result in the undertreatment of a cohort of patients at significant risk of thromboembolism.

A new risk scoring system would have to be more reliable in identifying truly low-risk patients and minimising patients being denied anticoagulation while they remained at risk of stroke. The CHA_2_DS_2_VASc score [26] has been developed and is better at identifying truly low-risk patients while placing fewer patients into the intermediate group [27]. The CHA_2_DS_2_VASc score has now been well-validated in a number of cohorts [22] and has been found to perform significantly better than CHADS_2_ [28]. The CHA_2_DS_2_VASc score (see Table **2**) has been incorporated into major international guidelines [8].

Bleeding is the most feared adverse effect associated with anticoagulation. Many of the risk factors for bleeding are also risk factors for thromboembolism, so the limiting effect of bleeding risk on the prescription of antithrombotics means a number of patients are untreated despite clear indications for anticoagulation [29]. The HAS-BLED score [30] has been proposed as a simple tool to aid clinicians in undertaking a bleeding risk assessment (see Table **3**). 

The HAS-BLED score should prompt clinicians to consider the correctable risk factors for bleeding, such as labile INR or concomitant drugs. It allows for periodic reassessment of the bleeding risk and considers the individual quality of INR control in each patient [31]. The HAS-BLED score has now been incorporated into guidelines on the management of AF [8] following its validation in various large cohorts of patients [32] and its favourable performance when compared to alternative bleeding risk scores [33].

## THE CASE FOR WARFARIN

Warfarin exerts its anticoagulant effect by interfering with the cyclic interconversion of vitamin K and its epoxide. Carboxylation of clotting factors by vitamin K is required for them to be biologically active [34], therefore when warfarin inhibits this process it results in the liver’s synthesis of ineffective coagulants [35]. 

Warfarin’s efficacy in the prevention of stroke in AF was irrefutably proved in a clutch of randomised control trials in the eighties and nineties [36-39]. Data from an initial analysis of five trials [40] showed that warfarin gave a 68% risk reduction in stroke compared to placebo. This evidence encouragingly led to an increase in the use of warfarin over the subsequent decade, which was accompanied by a decrease in the rate of ischaemic strokes [41]. Two recent meta-analyses showed that warfarin was far superior to aspirin for the reduction of ischaemic strokes [42] with a 40% reduction in strokes when compared to aspirin [43]. Warfarin has also been proven to reduce all-cause mortality by 26% in AF [43]. Conversely, when meta-analysis of antiplatelet therapy is restricted to aspirin only there is no significant effect on mortality and the reduction in stroke incidence was also a non-significant 19% [43]. A Japanese trial showed no difference between aspirin and the control group for thromboembolism, even amongst low risk patients, with a trend towards more bleeding with aspirin [24]. Aspirin was also ineffective at preventing severe strokes and was not beneficial for elderly patients. A randomised control trial dedicated to evaluating stroke prevention in elderly patients with atrial fibrillation also showed the superiority of warfarin, with no difference between warfarin and aspirin for major bleeding or intracranial haemorrhage [44]. 

A study in 2006 was undertaken with the intention of proving aspirin and clopidogrel in combination as non-inferior to warfarin for the prevention of thromboembolism in AF [45]. The trial was stopped early due to the clear superiority of warfarin over dual anti-platelet therapy. Furthermore, the rates of bleeding in the two groups were very similar (2.4% per annum for clopidogrel and aspirin vs. 2.2% per annum for warfarin). The evidence proves that antiplatelet therapy is an inferior option when compared to warfarin for thromboprophylaxis against stroke, and the comparable rates of haemorrhage mean it would not be an acceptable therapeutic strategy in patients deemed at too great a risk of bleeding to receive anticoagulation.

The optimal INR for patients with AF on warfarin for protection against thromboembolism has been established as 2.0-3.0 [46]. INR below 2.0 increases the risk for thromboembolism, whereas INR above 3.0 increases the risk of haemorrhage. The benefit of warfarin is dependent on the amount of time that patients spend in this optimal INR window [47]. The proportion of time spent with a therapeutic INR is referred to as the time in therapeutic range (TTR) and it is linked to most major outcome measures for AF. As well as thromboembolism the TTR also affects the rates of major haemorrhage, myocardial infarction and all-cause mortality [48]. The TTR is the best indicator of the quality of INR control and small improvements in TTR are associated with significant benefits [49], with very low TTR potentially obviating any benefit of anticoagulation. Self-monitoring can improve the quality of INR control [50] and may bring the TTR closer to that achieved in clinical trials [51]. 

## THE CASE AGAINST WARFARIN

While the efficacy of warfarin is unequivocal [52], the limitations of this inconvenient drug [53] have meant that clinicians and patients alike have had apprehensions preventing its universal uptake [17, 54]. Warfarin has a slow onset and offset of action and a narrow therapeutic window. There are wide inter-individual variations in dose requirements, primarily due to polymorphisms of genes that encode for the vitamin-K epoxide reductase enzyme and CYP2C9 enzyme [55]. Other drugs can interfere with the pharmacokinetics of warfarin by reducing gastrointestinal absorption or disrupting metabolic clearance [56]. Drugs also disrupt the pharmacodynamics of warfarin by inhibiting synthesis or increasing clearance of vitamin K-dependent clotting factors. Dietary intake of vitamin K can also exert an influence on the anticoagulant effect of warfarin [57]. Due to all of these factors (see Table **4**), warfarin requires frequent laboratory monitoring of its coagulation effect and subsequent dose alterations. This necessitates frequent clinic attendance with a consequential increase in healthcare costs and patient inconvenience.

The limitations associated with warfarin inform many of the characteristics that are sought by a successful novel oral anticoagulant (see Table **5**).

A new agent must have been shown to reliably perform as well as warfarin in randomised control trials, and be available in oral formulation for its easy application in the outpatient setting. The new agents should be free of severe adverse effects (ximelegatran was the first available novel oral anticoagulant but had to be withdrawn due to hepatotoxicity [58]). To represent a viable alternative to warfarin, new drugs should circumvent many of the limitations associated with warfarin that necessitate regular coagulation monitoring. They should therefore possess fixed dose regimens, wide therapeutic windows, low propensity for food and drug interactions, predictable pharmacokinetics and pharmacodynamics with little inter and intra patient variability. 

## NOVEL ORAL ANTICOAGULANTS 

The last few years have seen the emergence of several new oral anticoagulants which are poised to entire routine clinical practice and have the potential to offer effective thromboprophylaxis against stroke for a subset of patients who are not receiving anticoagulation despite their high-risk of thromboembolism. These new agents have more predictable pharmacokinetic and pharmacodynamics properties than warfarin, with their key attributes summarised in (Table **6**). The new drugs may eliminate the need for an initial two-drug regimen as they reach maximal effect within a few hours. The does responses are predictable and they have not been shown to have numerous significant drug or dietary interactions. None of these drugs require routine monitoring of coagulation.

**Fig. (1):**
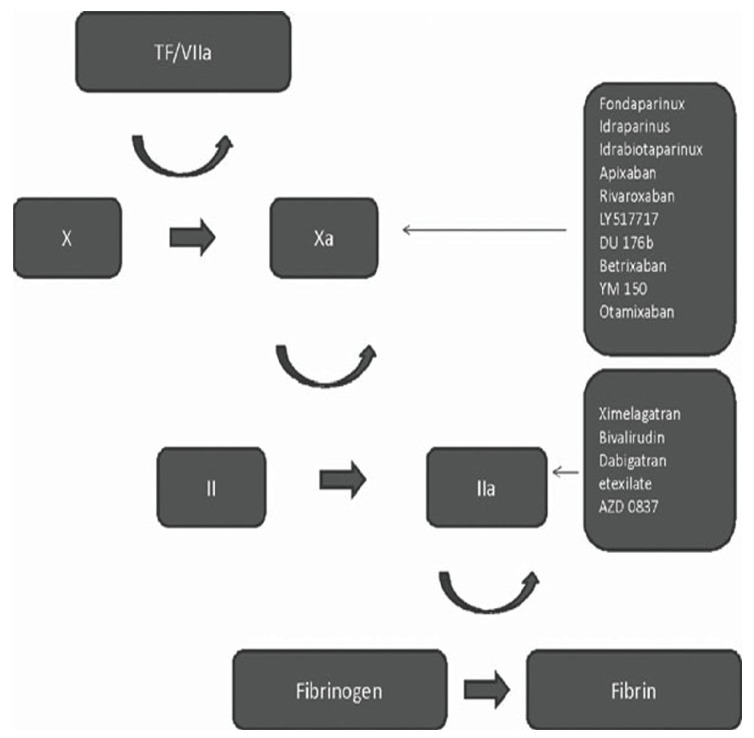
Sites of action of new anticoagulants [59].

While several potential targets for new anticoagulants have been identified (see Fig. **1**) [9], the two principal classes of agents available are direct thrombin inhibitors (dabigatran) and factor Xa inhibitors (rivaroxaban, apixaban). Factor Xa inhibition has more coagulation-specific effects, whereas direct thrombin inhibition may have beneficial effects outside of the coagulation cascade [32].

## DABIGATRAN

Dabigatran etexilate is an oral prodrug which is converted to the active compound (dabigatran) in the liver [60]. Dabigatran is a competitive, direct and reversible inhibitor of thrombin [61], exerting an effect on both clot-bound and free thrombin. The onset of action is fast with dabigatran (peak 0.5–4 hours), the half-life is 17 hours with multiple doses [62], and the main mode of elimination is *via* the kidneys [63]. 

RE-LY [64] was a large randomised controlled trial of over 18000 patients using PROBE design where dabigatran was compared with warfarin. Patients with nonvalvular AF and a CHADS_2_ score of 1 or higher were included or who were older than 65 years with coronary artery disease (see Table **7**) [65]. Two doses of dabigatran (110mg BD and 150mg BD) were compared to dose-adjusted warfarin. The primary efficacy outcome was stroke or systemic embolism. The low-dose of dabigatran was equivalent to warfarin for the prevention of stroke (RR 0.91; p0.34) whereas the high-dose of dabigatran was superior with a 34% reduction in stroke or systemic embolism (p<0.001). There was a trend towards a reduction in all-cause mortality with dabigatran, which approached significance in the high-dose dabigatran group.

Major bleeding was the primary safety outcome (defined as a reduction in haemoglobin level of 2 g/dl, transfusion requiring at least 2 units of blood, or symptomatic bleeding in a critical area or organ). Dabigatran 110mg was superior to warfarin with a 20% reduction in major bleeding (p=0.003), whereas dabigatran 150mg led to similar rates of major bleeding as warfarin (p=0.031). Both doses of dabigatran caused significantly less intracranial bleeding than did warfarin.

Warfarin was better tolerated than dabigatran: discontinuation rates were 21% for dabigatran 110 mg, 21% for dabigatran 150 mg, and 17% for warfarin at the end of the second year of the trial (p<0.001 for dabigatran vs warfarin). The main driver for drug discontinuation in the dabigatran arm was dyspepsia, felt likely to be due to the tartaric acid core of the compound.

Dabigatran 150 mg was found to have an increased rate of myocardial infarction (0.74%) when compared with warfarin (0.53%/year), although this effect did not reach statistical significance (RR 1.38, 95% CI 1–1.91, p=0.04). Warfarin has been proven in the past to protect against myocardial infarction [66], and it is eminently possible that the discrepancy in rates of infarction is driven primarily by warfarin’s protective properties rather than an intrinsic risk of dabigatran therapy. An analysis of subsequently discovered events in the RE-LY trial found this signal for increased myocardial infarction to be even less pronounced.

## RIVAROXOBAN

Rivaroxaban is an oral, reversible, direct factor Xa inhibitor [67]. It has high oral bioavailability [68], is rapidly absorbed with a half-life of 9-12 hours [69, 70] and a fast onset of action with maximal concentrations reached between 2 and 4 hours. There are multiple modes of elimination, with one third of the drug renally cleared and two-thirds being cleared extra-renally (predominantly in the liver) [71]. The pharmacokinetics of rivaroxaban are dose-proportional and unaffected by gender, body weight or extremes of age [72, 73]. 

ROCKET-AF [74] was a phase III, randomised, double-blind, event-driven noninferiority trial with over 14,000 patients comparing rivaroxaban with warfarin in nonvalvular AF (at least two documented episodes) and a history of stroke, TIA, or non-CNS embolism or at least two independent risk factors for future stroke. The patient population was high-risk, with the majority of patients having a CHADS_2_ score of 3 or greater. In contrast to RE-LY, this was a double-blind double-dummy trial with sham INRs. Patients were randomised to rivaroxaban 20 mg once daily (or 15 mg once daily in patients with moderate renal impairment), or dose-adjusted warfarin. The primary end point was stroke or systemic embolism. Rivaroxaban was non-inferior to warfarin for the prevention of stroke: HR 0.79, 95% CI 0.66–0.96, p<0.001 for noninferiority. The intention-to-treat analysis could not demonstrate the superiority of rivaroxaban: HR 0.88, 95% CI 0.74–1.03, p=0.117 for superiority. Superiority was, however, demonstrated in the per-protocol analysis of patients who continued to receive treatment for the 40-month trial period with a 21% reduction in stroke or systemic embolism (HR 0.79, 95% CI 0.65–0.95, p=0.015).

Bleeding was similar overall in the two groups (HR 1.03, 95% CI 0.96–1.11, p=0.442) although rivaroxaban led to significantly less fatal bleeding and intracranial haemorrhage. Paradoxically, more patients in the rivaroxaban arm suffered a haemoglobin decrease of 2 g/dl or more or required a blood transfusion. The two drugs were equally well-tolerated.

### Apixaban

Similarly to rivaroxaban, apixaban is an oral, selective, reversible direct factor Xa inhibitor [75] with high oral bioavailability [76] and a rapid onset of action [60]. It has a half-life of 12 hours [77]. There is only a minimal contribution of the kidneys to the elimination of the drug, with the majority being cleared in faeces [76]. 

The AVERROES trial [78] evaluated apixaban against aspirin for the prevention of stroke in patients deemed unsuitable for warfarin. The study was ended prematurely due to the clear superiority of apixaban. Apixaban was associated with a 55% reduction in the primary endpoint of stroke or systemic embolism (p<0.001), with no increase in bleeding compared to aspirin (HR 1.13, 95% CI 0.74–1.75, p=0.57). Furthermore, apixaban was better-tolerated than aspirin.

The ARISTOTLE study [79] was a large randomised trial comparing apixaban 5 mg BD versus dose-titrated warfarin in over 18,000 patients [80]. Similarly to ROCKET-AF this was a double-blind, double-dummy study with sham INRs. The primary outcome was stroke (either ischaemic or haemorrhagic) or systemic embolism, and the trial was designed to test for noninferiority. Apixaban was found to be superior to warfarin for the primary end-point of all-cause stroke or systemic embolism: HR 0.79; 95% CI 0.66-0.95; p=0.01 for superiority). This was primarily driven by a reduction in haemorrhagic stroke, as the rates of ischaemic stroke were equivalent in the two groups. Haemorrhagic stroke was 0.24% per year in the apixaban group versus 0.47% per year in the warfarin group (hazard ratio, 0.51; 95% CI, 0.35 to 0.75; P<0.001). Apixaban was the only new anticoagulant to demonstrate a benefit with regards to all-cause mortality compared to warfarin (HR 0.89; 95% CI, 0.80 to 0.99; P=0.047). Apixaban was associated with a 31% reduction in major bleeding compared to warfarin (p<0.001). Apixaban was found to be better tolerated than warfarin.

## ROLE IN MANAGEMENT

There are several similarities that can be drawn from the three major phase III trials published which compare warfarin to novel oral anticoagulants. All three agents significantly reduced the rates of haemorrhagic stroke, and this was the primary driver in the reductions for the primary endpoints in all trials. Only dabigatran 150mg BD was shown to significantly reduce the risk of ischaemic stroke. All three drugs also demonstrate positive bleeding profiles (esp. intracranial haemorrhage) when compared to warfarin. Apixaban is the only new agent which demonstrated a statistically significant reduction in all-cause mortality compared to warfarin, although dabigatran 150mg BD and rivaroxaban also showed a trend towards reduction in the risk of death. 

There are also differences between the three trials in design and study population. The RE-LY trial was an open trial, with a PROBE design. It is difficult to offer conclusive deductions on the new agents based on the differences in the results from the three distinct trials. ROCKET-AF had a generally higher risk patient group, with 86% of the total population possessing a CHADS_2_ score of 3 or higher. RE-LY and ARISTOTLE had significantly more low-risk patients (32% of patients in RE-LY and 34% in ARISTOTLE had a CHADS_2_ score of 0-1, compared to <1% for ROCKET-AF). ROCKET-AF, however, had poorer quality of INR control with mean TTR of 55% whereas the mean TTR was 62% in ARISTOTLE and 64% in RE-LY. Meaningful comparisons between the agents will only be achieved in head-to-head trials. 

Thus far only dabigatran and rivaroxaban has been approved by the FDA, and dabigatran has been incorporated into guidelines in Europe [8], the US [81] and Canada [82]. 

## LIMITATIONS OF NEW AGENTS

As well as appreciating the great potential these new agents have in improving our ability to effectively treat AF patients with thromboprophylaxis, we must also appreciate their potential pitfalls. Dabigatran and apixaban require twice-daily dosing, which favours forgetfulness and raises the possibility of more missed doses. Due to the short half-lives of these drugs, omitted doses may place the patient in a prothrombotic state and increase the risk of thromboembolism. This effect may be compounded by the fact that routine coagulation monitoring is unnecessary: regular INR checks permit clinicians to assess the compliance of a patient, as well as dispense health education and affirm how crucial it is for patients to adhere to their anticoagulation. There is a danger that adoption of the novel oral anticoagulants may lead to the situation seen with other cardiovascular drugs, where poor adherence is a widely-encountered problem [83-85]. 

Dabigatran is contraindicated in patients with severe renal insufficiency and must be used in caution in patients with moderate renal dysfunction (as must rivaroxaban). The dose of dabigatran approved by the FDA in renal impairment was never actually studied in the RE-LY trial [86]. The rates of stroke and thromboembolism were proportionally increased in patients with renal insufficiency in a subgroup analysis of the RE-LY trial. 

As well as the appropriate dose reductions, a position paper from the Italian Federation of Thrombosis Centres [87] advises on 12-monthly and 6-monthly monitoring of renal function in patients with mild and moderate renal impairment respectively. Clinicians are also advised to be vigilant when interpreting the serum creatinine of elderly patients, who may have misleading results owing to a decline in muscle mass.

The RE-LY trial also highlighted dabigatran’s propensity to cause dyspepsia and other gastrointestinal upset, likely caused by the tartaric acid core of the dabigatran core which leads to an acidic microenvironment. The Italian position paper suggests patients with recurrent dyspepsia should remain on warfarin, although rivaroxaban and apixaban may be potentially effective therapeutic strategies for patients with GI dysfunction who are truly unable to take warfarin. The Italian consensus paper also recommends patients with a previous myocardial infarction remain on warfarin and do not receive dabigatran. This stems from the RE-LY trials reporting of an increase in the number of myocardial infarctions in the dabigatran group – although this trend did not reach statistical significance and was less pronounced in a report of supplementary findings from the RE-LY trial [88]. Nonetheless, in view of warfarin’s proven efficacy as secondary prevention against myocardial infarction, it remains a sensible first choice in this patient group.

Although dabigatran and the other novel oral anticoagulants do not require routine monitoring of coagulation effect, there are certain situations when a laboratory measure of anticoagulant effect would be needed (emergency presentation with thrombosis or haemorrhage, renal or liver failure, suspected adverse drug interaction, intentional anticoagulant overdose and other situations that call for immediate reversal of anticoagulation). The aPTT may offer a qualitative measure of the anticoagulant effect of direct thrombin inhibitors [89], it is not suitable as a precise measure [90] owing to the flattening of the concentration-response curve at higher concentrations [91]. The prothrombin time or INR is not affected to a great degree by dabigatran [92] and therefore the INR is unsuitable as a measure of the coagulation effect of dabigatran (the responsiveness is poor, with INRs of 2.0 or less obtained with supratherapeutic concentrations of dabigatran). The thrombin clotting time (TT) provides a direct measure of the activity of dabigatran. There is a linear dose-responsiveness curve but the sensitivity of the test is excessive [92]. A specific diluted TT assay for the measurement of dabigatran levels is currently in development and has favourable results in a paediatric population [93]. Until such an assay is commercially available, the best test to assess the coagulant effect of dabigatran in the Ecarin clotting time (ECT). The ECT is a specific assay for thrombin generation and provides a direct measure of the activity of dabigatran with good linearity and excellent responsiveness [94]. Rivaroxaban activity is best measured by an index to convert prothrombin time into an INR-rivaroxaban measure, with good linearity and acceptable responsiveness [95]. The responsiveness of the APTT is poor, and anti-FXa assays are not yet readily available [95]. Whichever test is utilised to assess coagulation activity, it is prudent practice to perform the test after initiation and establishment of the drug in order that a reference value for each patient is available for future comparisons in the event of adverse events.

There are situations when anticoagulation needs reversal, generally either in the emergency setting due to bleeding or for planned elective surgery. Warfarin has an established antidote in the form of vitamin K, but it should be remembered that even after intravenous administration of vitamin K then INR takes several hours to normalise [96]. The novel oral anticoagulants lack a specific antidote (although a phase IV study is investigating potential reversal options for dabigatran [97] and work is being done on an antidote for factor Xa inhibitors [98]) although they exert a shorter duration of anticoagulant effect than warfarin.

In patients with normal renal function dabigatran can be discontinued 24 hours prior to surgery, increasing to 2-4 days if the patient has impaired renal function or is deemed at high risk for bleeding [91]. A laboratory measure of anticoagulant activity is advised in patients with renal dysfunction or elevated bleeding risk. There are no randomised control trials evaluating perioperative out-comes in dabigatran-treated patients requiring surgery. The overall decision regarding how to bridge anticoagulant therapy at the time of surgery requires the judgment of an experienced clinician who must take into account the type of surgery being undertaken, the patient’s relative risk of bleeding and thromboembolism, the renal function and the quality of anticoagulation.

When bleeding is encountered in patients taking a novel antithrombotic agent, first stop the drug as the relatively short half-lives ensure the blood levels of the drug will fall rapidly. For predominantly renally-excreted drugs such as dabigatran, it is crucial to ensure an adequate level of diuresis. Supportive measures should be given as with all cases of bleeding, including transfusion of packed red cells or fresh frozen plasma as the clinical scenario dictates. If life-threatening bleeding is encountered and supportive measures are insufficient, recombinant factor VII or prothrombin complex concentrates [99] may have potential according to preclinical studies – although more data are required. Dabigatran can also be successfully adsorbed by activated charcoal [100] or removed by haemodialysis [101] in cases of overdose or where rapid reversal of anticoagulation is mandatory. 

Clinicians are well aware of the limitations of warfarin and the need for alternative therapies. We must also now be aware of the shortcomings of the new agents (see Table **8**) and recognise the factors and nuances pertinent to each clinical encounter which will favour differing antithrombotic strategies.

Although warfarin is among the top drugs frequently associated with serious adverse events requiring admission [102], not all patients will benefit from switching to a different drug. Those who are well-established on warfarin and have good INR control with high TTR ought to remain on warfarin; similarly it is probably unwise to switch to dabigatran in patients with dyspepsia or who have suffered a previous myocardial infarction if they are tolerant of and compliant with warfarin. However, patients with a history of cerebral haemorrhage ought to be considered for transition from warfarin to dabigatran in view of the lower rates of intracranial bleeding. Patients who are unwilling or unable to comply with INR monitoring would also likely benefit from treatment with a novel oral anticoagulant. 

It is vital that as clinicians we collaborate with our patients when determining the most suitable strategy for stroke prevention. A good level of understanding on the behalf of the patients translates to better clinical outcomes [103]. As a group, it seems physicians tend to overestimate the burden of anticoagulant monitoring [104] whereas patients would generally reasonably accept an increase in inconvenience to avoid a serious event like a stroke [105]. Patients taking a novel agent must be fully counselled that we are still inexperienced in the use of these drugs and more time and research is required to glean the full safety profile of these treatments. Our knowledge of and confidence in these therapies will be enhanced by longer-term trials focusing on patient safety [106]. 

## DRONEDARONE

Antiarrhythmic therapy has traditionally not shown a significant beneficial impact on cardiovascular complications. The AFFIRM trial [107] demonstrated there was no benefit with rhythm control when compared to rate control for the endpoints of cardiac or vascular mortality. The RACE II trial [108] also proved that lenient rate control was easier to achieve in patients with permanent AF and as effective.

Therefore until the advent of dronedarone, there was no cardiovascular benefit to be derived from rhythm control. Dronedarone is an amiodarone analogue that differs structurally from amiodarone in that the iodine moiety was removed and a methane-sulfonyl group was added [109] to shorten its half-life and reduce the adverse effects. 

The ATHENA trial [110] was a placebo-controlled, double-blind trial to assess the efficacy of dronedarone for the prevention of cardiovascular hospitalisation or death from any cause in patients with AF. The dronedarone group demonstrated a statistically significant reduction in all-cause mortality or cardiovascular hospitalisation (HR 0.76; 0.69-0.84; p<0.001). A further analysis of ATHENA [111] demonstrated that dronedarone reduced the risk of stroke from 1.8% per year to 1.2% per year (HR0.66; 0.46-0.96; p=0.027). This effect was consistent regardless of whether patients were taking oral anticoagulation, and was most pronounced in higher-risk patients with CHADS_2_ scores ≥3. There was also a 31% reduction in stroke-related hospitalisations. 

The PALLAS trial was intended to enrol 10,800 patients with permanent AF and randomise them to dronedarone or placebo in an attempt to better understand the benefit of dronedarone. The composite endpoints included: 1) first stroke, systemic embolism, myocardial infarction, cardiovascular death; 2) first unplanned cardiovascular admission, death from any cause. After over 3000 patients had been enrolled, the trial was prematurely halted due to a two-fold increase in cardiovascular events in the dronedarone group (death, stroke, heart failure hospitalisation) [112]. Thus the current role of dronedarone in the prevention of stroke in AF is unclear, and more long-term and robust data are required to inform us whether novel antiarrhythmic therapies will significantly impact on stroke prevention.

## CONCLUSIONS

A decade of intensive research into AF has resulted in major progress in the management of this disorder. Contemporary well-validated stroke risk factor schemes (CHA_2_DS_2_-VASc) now permit rapid but comprehensive evaluation of a patient’s risk for thromboembolism. We are better equipped to accurately identify low-risk patients who do not need to be burdened with antithrombotic therapy, and are clearer that the majority of AF patients (those with at least one other stroke risk factor) require formal oral anticoagulation. Aspirin has been proven to be an inferior option to anticoagulation, and is not free of bleeding risk. We also have the simple validated tools to evaluate a patient’s risk of haemorrhage (HAS-BLED). The interplay of these three factors (stroke risk scoring, bleeding risk scoring, the limitations of aspirin) should mean fewer patients are undertreated with an ineffective therapy and more receive appropriate protection from thromboembolism.

The emergence of a clutch of new oral anticoagulants should further shift the landscape towards improved stroke prevention in atrial fibrillation [113]. These agents share common properties that mean they can successfully negotiate many of the hurdles to oral anticoagulation generated by warfarin’s limitations. More patients should now be receiving oral anticoagulation as for the first time in six decades warfarin is no longer the only effective therapeutic option. Long-term data on the safety, efficacy and cost-effectiveness of the new oral anticoagulants is required to properly assess how they compare to warfarin; and head-to-head trials between the new drugs will be required before any meaningful conclusions can be drawn to determine how they perform against each other. Clinicians using the new anticoagulants must come to terms with a new set of limitations distinct from those of warfarin. Monitoring, reversal, and perioperative management are all areas which require further investigation to enhance our ability to safely and effectively utilise the new agents. We are entering a new era for stroke prevention in AF, but there is still much to learn. 

## Figures and Tables

**Table 1.** The CHADS_2_ Score for Stroke Risk in AF

Risk factor	Score
Congestive heart failure	1
Hypertension	1
Age ≥ 75 years	1
Diabetes mellitus	1
Stroke/TIA/TE	2
Maximum score	6

**Table 2.** The CHA_2_DS_2_-VASc Score for Risk of Stroke in Nonvalvular AF

Risk Factor	Score
Congestive cardiac failure	1
Hypertension	1
Age ≥ 75	2
Diabetes mellitus	1
Stroke/TIA/thromboembolism	1
Vascular disease	1
Age 65-74	1
Female sex	1
Maximum score	9

A score of 0 indicates low risk; 1 indicates moderate risk; ≥2 indicates high risk. Congestive cardiac failure is defined as left ventricular ejection fraction ≤40%. Hypertension is defined as blood pressure consistently above 140/90 mmHg or treated hypertension on medication. Vascular disease is defined as previous myocardial infarction, peripheral arterial disease or aortic plaque.

**Table 3.** The HAS-BLED Score for Bleeding Risk

Risk Factor	Score
Hypertension	1
Abnormal renal/liver function	1 or 2
Stroke	1
Bleeding tendency	1
Labile INR	1
Age >65	1

A score of 0-2 indicates low risk of bleeding; ≥3 indicates high risk of bleeding Hypertension is defined as a systolic blood pressure > 160 mmHg 1 point is awarded for each of abnormal renal or liver function, and drugs or alcohol.

**Table 4.** Limitations of Warfarin

Frequent monitoring necessitating regular clinic attendance Narrow therapeutic window Slow onset and offset of action, requiring 3-6 days to reach therapeutic levels Long half-life Numerous drug and dietary interactions Genetic polymorphisms exist which confer increased sensitivity or resistance to warfarin Unpredictable pharmacodynamics and pharmacokinetics leading to inter and intra-individual variability in dose and metabolism

**Table 5.** Characteristics of the Ideal Anticoagulant

Equivalent efficacy to warfarin at least Predictable response Wide therapeutic window Low inter and intra-patient variability Fixed oral dosing Low potential for drug and dietary interactions No need for regular coagulation monitoring Fast onset and offset of action Low incidence and severity of adverse effects

**Table 6.** Pharmacokinetic and Pharmacodynamics Properties of the Novel Anticoagulants

	Dabigatran	Rivaroxaban	Apixaban
Mechanism of action	Direct thrombin inhibitor	Direct factor Xa inhibitor	Direct factor Xa inhibitor
Prodrug	Double prodrug	No	No
Dosing frequency	Twice daily	Once daily	Twice daily
Bioavailability %	6.5	50	80
T_max_	2 hours	2-4 hours	3 hours
Half-life	17 hours with multiple doses, 7-9 hours with single doses	9 hours in healthy subjects, 12 hours in elderly subjects	12 hours
Mode of excretion	80% cleared renally	One-third cleared renally, two-thirds metabolised by the liver	70% cleared in faeces, 25% cleared renally
Age effect	Affects pharmacokinetic parameters	No	No
Drug interactions	Interaction with aspirin at high doses	None reported	None reported

**Table 7.** Outcomes of the RE-LY Trial

Outcome	Dabigatran 110mg	Dabigatran 150mg	Warfarin	RR Dabiagtran 110mg Versus Warfarin	RR Dabigatran 150mg Versus Warfarin
Stroke or systemic embolism	1.53	1.11	1.69	0.91; 0.74-1.11 (p=0.34)	0.66; 0.53-0.82 (p<0.001)
Major bleed	2.71	3.11	3.36	0.80; 0.69-0.93 (p=0.003)	0.93; 0.81-1.07 (p=0.31)
Intracranial haemorrhage	0.23	0.3	0.74	0.31 (p<0.001)	0.40 (p<0.001)
GI haemorrhage	1.12	1.51	1.02	1.10; 0.86-1.41 (p=0.43)	1.50; 1.19-1.89 (p<0.001)
Life-threatening haemorrhage	1.22	1.45	1.8	0.68; 0.55-0.83 (p<0.001)	0.81; 0.66-0.99 (p=0.04)
Acute myocardial infarction	0.82	0.81	0.64	1.29; 0.96-1.75 (p=0.09)	1.27; 0.94-1.71 (p= 0.12)
Mortality	3.75	3.64	4.13	0.91; 0.80-1.03 (p=0.13)	0.88; 0.77-1.00 (p = 0.051)

**Table 8.** Potential Limitations of New Anticoagulants

No known antidote Lack of validated tests to monitor anticoagulant effect It is difficult to assess compliance A method of anticoagulant bridging prior to surgery has not been established Unknown long-term safety profile Unknown true cost-effectiveness compared to warfarin No head-to-head studies of new agents Dabigatran and apixaban require twice daily dosing, which may promote forgetfulness Dabigatran has been associated with GI side-effects
